# Mica Can Alleviate TNBS-Induced Colitis in Mice by Reducing Angiotensin II and IL-17A and Increasing Angiotensin-Converting Enzyme 2, Angiotensin 1-7, and IL-10

**DOI:** 10.1155/2020/3070345

**Published:** 2020-10-10

**Authors:** Mengdie Shen, Bibi Zhang, Mengyao Wang, Li'na Meng, Bin Lv

**Affiliations:** ^1^Department of Internal Medicine, Women's Hospital, Zhejiang University School of Medicine, Hangzhou, 310006 Zhejiang Province, China; ^2^Department of Gastroenterology, First Affiliated Hospital of Zhejiang Chinese Medical University, Hangzhou City, Zhejiang Province 310006, China; ^3^Key Laboratory of Digestive Pathophysiology of Zhejiang Province, China; ^4^Institute of Digestive Pathophysiology, Zhejiang Chinese Medical University, China

## Abstract

**Aim:**

To explore the treatment effect of mica on 2,4,6-trinitrobenzenesulfonic acid solution- (TNBS-) induced colitis in mice.

**Materials and Methods:**

Thirty male BALB/C mice were randomly divided into the control group, the TNBS group, and the mica group. Control mice were treated with saline solution. Experimental colitis was induced by TNBS (250 mg/kg/d) in the TNBS group and the mica group. After modeling, the mica group was treated with mica (180 mg/kg/d) for 3 days, while the TNBS group continued the treatment with TNBS. All solutions were injected intrarectally. During treatment, body weight and mice activity were monitored daily. After treatment, the colon tissues of mice were collected; angiotensin II (Ang II), angiotensin-converting enzyme 2 (ACE2), angiotensin 1-7 (Ang (1-7)), IL-17A, and IL-10 expression was analyzed by ELISA and immunohistochemistry.

**Results:**

Food intake, activity, and body weight gradually decreased in the TNBS group compared to the control group and the mica group (all *P* < 0.05). Also, black stool adhesion in the anus and thin and bloody stool were observed in the TNBS group, but not in the other two groups. Moreover, the expression of Ang II, ACE2, Ang (1-7), IL-17A, and IL-10 in the TNBS group increased compared to that in the control group. Compared to the TNBS group, ACE2, Ang (1-7), and IL-10 in the mica group increased, while Ang II and IL-17A decreased (all *P* < 0.05).

**Conclusion:**

Mica can alleviate TNBS-induced colitis in mice by regulating the inflammation process; it reduces Ang II and IL-17A and increases ACE2, IL-10, and Ang (1-7).

## 1. Introduction

Inflammatory bowel disease (IBD) is a chronic nonspecific inflammatory disease that affects the gastrointestinal tract. Over the past 20 years, the incidence of IBD in Asian countries, especially China, has shown a rapid increase [1]. In 2018, the standardized incidence of IBD in Daqing, a city in the west of Heilongjiang province, was 177/100,000, while it was only 3.14/100,000 in Zhongshan city, Guangdong [2]. IBD is characterized by chronic inflammation of your digestive tract. Early and rapid diagnosis of the disease and improvement of intestinal inflammation are critical steps in preventing further progression and improving prognosis. Yet, the exact etiology and pathogenesis of IBD remain unclear.

Traditional therapeutic drugs can improve symptoms but have no effect on the inflammatory process. The renin-angiotensin system (RAS) is an important regulating system, which participates in multiple inflammatory responses. RAS has a vital role in chronic inflammation and early inflammation. Angiotensin II (Ang II) is an essential active peptide of the RAS system [3]. Previous studies [4, 5] have found that Ang II is expressed in the colon tissues, where it participates in the process of intestinal inflammatory reaction and tissue damage [6]. Thus, it may have an important role in the occurrence of ulcerative colitis.

Mica is a kind of natural layered mineral crystal, which is used in the clinical treatment of various gastrointestinal diseases. Previous studies have shown that this mineral can promote the regeneration of gastrointestinal mucosal epithelial cells, maintain the mucosal barrier, and have an inhibitory effect on the inflammation [7]. The aim of this study was to explore the treatment effect of mica on TNBS-induced colitis in mice.

## 2. Materials and Methods

### 2.1. Animals

Thirty male BALB/C mice, 6-8 weeks old, weighing 20 ± 5 g, were purchased from the animal experimental center of Zhejiang Chinese Medical University (2008001664294). All the animals were housed in an environment with a temperature of 22-26°C, relative humidity of 50-60%, and a light/dark cycle of 12/12 h and fed with standard mouse diet. All animal studies (including the mouse euthanasia procedure) were done in compliance with the regulations and guidelines of Zhejiang Chinese Medical University institutional animal care and conducted according to the AAALAC and the IACUC guidelines.

### 2.2. Reagents

2,4,6-Trinitrobenzenesulfonic acid solution (no. P2297) was produced by Sigma Company in the United States. Mica microscopical particles were provided by the Department of Pharmacy, the First Affiliated Hospital of Zhejiang Chinese Medical University. Rabbit polygonal to anti-ACE2 antibody was from Abcam, UK; rabbit polygonal to anti-Ang II antibody from British Biorbyt, UK; rabbit polygonal to anti-Ang (1-7) from Cloud-Clone Corp., USA; and rabbit polygonal to anti-IL-10 from Abcam, UK. A mouse IL-17A ELISA Kit was purchased from Abcam, UK.

### 2.3. Grouping, Modeling, and Specimen Collection

Thirty male BALB/C mice were randomly divided into three groups (10 mice/group): control group, TNBS group, and mica group. Referring to the modeling method of Inokuchi et al. [8], 5% TNBS solution was mixed with anhydrous ethanol in equal volume, configured into a solution of 50 mg/ml, and enema was given once with the dose of 250 mg/kg.

Before modeling, mice were fasted for 24 h. Then, mice were anesthetized using 1% pentobarbital, and the paraffin oil was adequately lubricated. The silicone tube with a diameter of about 2 mm was then inserted into the anus about 3.5 cm; after which, the TNBS ethanol solution 250 mg/kg (for the TNBS group and the mica group) and saline enema with 1 ml/100 g/d (control group) were slowly injected into the colon. After the silicone tube was pulled out, the anus was pinched, and mice were inverted for another 1 min before being put into the squirrel cage. On days 2, 3, and 4, the control group and the TNBS group were given a normal saline enema, and the mica group received mica enema with 180 mg/kg/d (referring to a previously described approach [9]). On day 5, all the mice were sacrificed, and colon specimens (including the intestinal segment from the anus) were collected.

Tissue was rinsed with normal saline; the damage of the intestinal mucosa was analyzed using a microscope. In the TNBS group and the mica group, the most apparent lesions were collected; in the control group, colon tissue was collected at a distance of 3-4 cm from the anus. Two segments were taken for each mouse. The first was fixed in 10% formaldehyde and embedded in paraffin, followed by hematoxylin-eosin staining and immunohistochemistry. The other section was placed into a cryopreservation tube and stored in a refrigerator at -80°C for inspection.

### 2.4. General Condition

The animal weight was analyzed daily. The weight was calculated according to the beginning of the experiment. In addition, fecal characteristics (normal, loose, loose stool), fecal bleeding (occult blood or visual blood stools), activity, and feeding were also monitored on a daily basis. According to the scoring method proposed by Murano et al. [10], the disease activity index was the sum of the three scores of weight loss rate, fecal characteristics, and hematochezia (Table 1). A fecal occult blood test was conducted using tetramethylbenzidine.

### 2.5. Analysis and Scoring of Intestinal Damage

The degree of colonic mucosal damage and inflammation was observed by the score standard of macroscopic damage, referring to Murano et al. [10]. According to the histological damage score method of Dieleman et al. [11], the histological damage degree was revealed by the sum of inflammation, lesion depth, recess failure score, and lesion range score. The scoring criteria are shown in Tables 2 and 3.

### 2.6. ELISA Detection of IL-17A in the Colon

The level of IL-17A in the colon tissue was determined by Mouse IL-17A ELISA Kit (Abcam, UK) and ELISA El 800 readers (BioTek Instruments, USA), following the manufacturer's instruction.

### 2.7. Immunohistochemical Detection of Ang II, ACE2, Ang (1-7), and IL-10 in Colon Tissue

The colon tissues were fixed with 10% formalin, dehydrated, and cut into 5 *μ*m thickness section. Samples were then subjected to immunohistochemical SP staining. The number of staining-positive cells in more than 200 cells was counted and converted to a positive market index, which was calculated using the following formula: positive index (%) = the number of positive cells in the total field/the number of cells in the field 100%. Staining intensity was analyzed using an immune response score (IRS): 0 for colorless, 1 for light yellow, 2 for brownish yellow, and 3 for brown. The percentage score of positive cells was calculated as follows: 1 was divided into positive cells ≤ 10%, 2 into 11%-50%, 3 into 51-75%, and 4 into >75%. The product of staining intensity and percentage of positive cells was a positive integral. A positive integral greater than 3 was considered to be immunoreactive.

### 2.8. Statistical Analysis

The measurement data conforming to the normal distribution were expressed as mean ± standard deviation, and the comparison between groups was performed by one-way analysis of variance. Pearson was used for correlation analysis. The statistical significance of all tests was defined as *P* < 0.05. All analysis was performed using IBM SPSS statistical software, version 22.0.

## 3. Results

### 3.1. Animal Condition and the Colon Morphology

**Figure 1:**
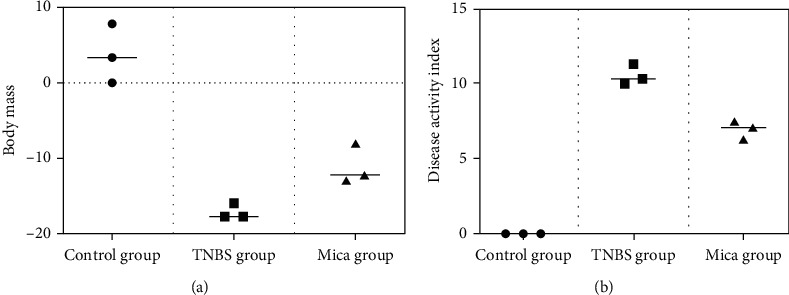
(a) Weight loss in each group and (b) disease activity index in each group.

The mice in the control group showed good activity, a slow weight increase, and a clean anal opening (Figure 1, Table 4). In the TNBS group, the intake, the amount of activity, and body weight gradually decreased (Figure 1, Table 4). In addition, the black stool adhesion in the anus, accompanied by thin stool, positive fecal occult blood, and some blood, was observed in the TNBS group. On the second day after modeling, one mouse died, and the dead mouse was dissected. Colon perforation was found about 4 cm from the anus, and feces were seen in the abdomen.

In the mica group, the weight loss was reduced, and the stool characterization was normal compared to the TNBS group (Figure 1, Table 4). In the mica group, one mouse died on day two. Pathological examination of the dead mice showed that the colon adhered to the surrounding tissues 3-4 cm from the anus.

The disease activity index of mice in the TNBS group significantly increased on the first day after modeling, reaching the highest score on the second day. The disease activity index of mice in the mica group was significantly lower than that in the TNBS group. The weight loss and disease activity index in each group are shown in Figure 1 and Tables 4–5.

### 3.2. Macroscopic Analysis and Scoring of the Colon Damage

The colonic mucosa of the control group was pale pink, smooth, and complete, with normal colonic length and uniform thickness. The mice in the TNBS group had hyperemia and edema in the colonic wall, significant erosion; multiple ulcerations of different sizes were observed in the intestinal segment 2-4 cm from the anus, and dark red unformed feces were observed in the intestinal cavity. The colonic damage caused by TNBS was alleviated in the mica group, which showed local hyperemia and scattered erosion.

The TNBS group had a significantly higher colonic macroscopic damage score compared to the control group (4.00 ± 0.89 vs. 0.00 ± 0.00, *P* < 0.01) and the mica group (2.00 ± 1.53 vs. 4.00 ± 0.89, *P* < 0.01). The mica group had higher colonic macroscopic damage score compared to the control group (2.00 ± 1.53 vs. 0.00 ± 0.00, *P* < 0.01).

### 3.3. Histological Analysis and Scoring of the Colon Damage

**Figure 2:**
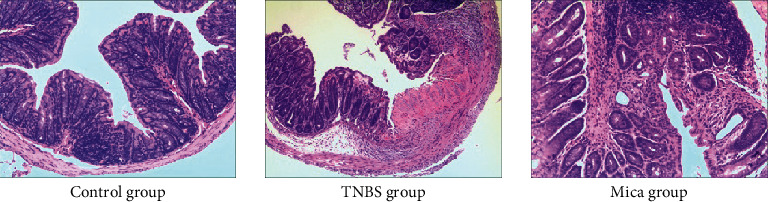
Histological analysis (HE staining) of the colon damage in different groups, ×200.

In the control group, the colonic mucosa was intact, and the cell structure was normal, and glands were normally arranged. In the TNBS group, the glandular arrangement was disordered, and some of the crypts were deformed or even disappeared, and the mucosa was ulcerated and necrotic. There were a large number of inflammatory cells infiltrated to the mucosa and submucosa. The damage in the mica group was significantly reduced compared with the TNBS group, and the damage degree of the structure of the crypt was alleviated. In addition, only a few inflammatory cells were found to be infiltrated into the mucosal layer. Histological analysis (HE staining) of the colon damage in different groups is shown in Figure 2.

The score of colonic histological damage in the TNBS group and the mica group was significantly higher compared to the control group (8.17 ± 2.99 vs. 1.33 ± 1.03, 3.83 ± 1.47 vs. 1.33 ± 1.03, *P* < 0.01). Yet, the score in the mica group was significantly lower compared to that in the TNBS group (3.83 ± 1.47 vs. 8.17 ± 2.99, *P* < 0.01) (Table 6).

### 3.4. The Expression of Ang II, ACE2, Ang (1-7), and IL-10 in the Colon

**Figure 3:**
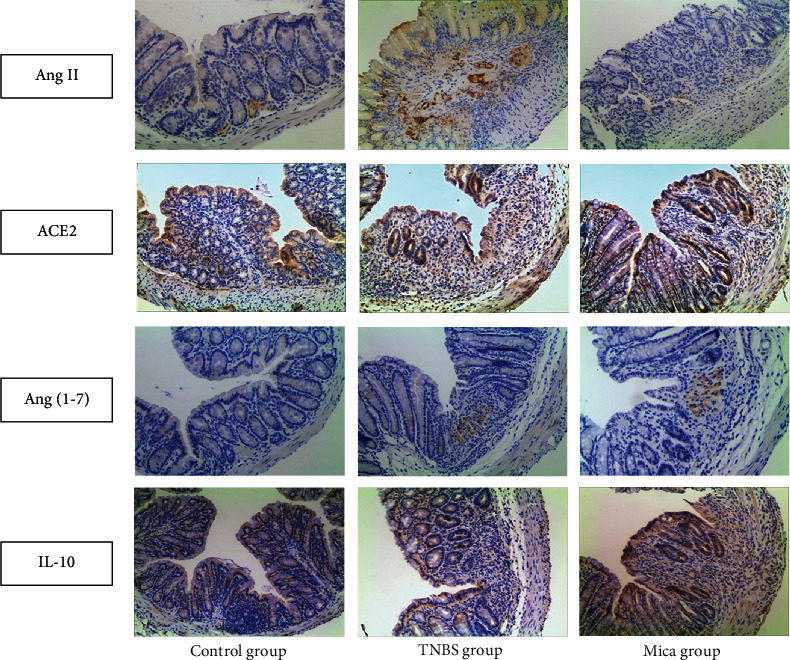
The expression of Ang II, ACE2, Ang (1-7), and IL-10 in different groups, ×200.

The expression of Ang II, ACE2, Ang (1-7), and IL-10 is shown in Figure 3 and Table 7.

The positive expression of Ang II was observed in the colonic vascular endothelial cytoplasm. It was also partially expressed in the mucosal epithelium and inflammatory cytoplasm. The expression of Ang II in the TNBS group was obviously higher compared to that in the control group (4.83 ± 2.11 vs. 2.16 ± 0.41, *P* < 0.01) and the mica group (2.33 ± 0.52 vs. 4.83 ± 2.11, *P* < 0.01).

The positive expression of ACE2 was mainly expressed in colonic mucosal epithelial cells and inflammatory cytoplasm. The expression of ACE2 in the TNBS group was obviously higher compared to that in the control group (3.50 ± 0.55 vs. 2.04 ± 0.29, *P* < 0.05). The expression of ACE2 further increased in the mica group compared to the TNBS group (5.13 ± 1.84 vs. 3.50 ± 0.55, *P* < 0.05).

The positive expression of Ang (1-7) was mainly found in colonic mucosal epithelial cells and inflammatory cytoplasm. The expression of Ang (1-7) in the colon tissues of the TNBS group was significantly higher compared to that of the control group (1.04 ± 0.56 vs. 0.13 ± 0.14, *P* < 0.05). The expression of Ang (1-7) further increased in the mica group compared to the TNBS group (2.0 ± 0.63 vs. 1.04 ± 0.56, *P* < 0.01).

The positive expression of IL-10 was mainly expressed in the cytoplasm of the colonic epithelial cells and inflammatory cells. The expression of IL-10 in the colon tissue of the TNBS group was significantly higher compared to that of the control group (4.88 ± 0.83 vs. 2.71 ± 0.78, *P* < 0.05). The expression of IL-10 further increased in the mica group compared to the TNBS group (9.04 ± 2.62 vs. 4.88 ± 0.83, *P* < 0.05).

### 3.5. ELISA Detection of IL-17A in the Colon

The level of IL-17A in homogenate supernatant of colonic mucosal tissues was determined by ELISA. The results showed that the expression of IL-17A in the TNBS group and the mica group was significantly increased compared to that in the control group (6.93 ± 0.44 vs. 0.65 ± 0.03, 2.63 ± 0.64 vs. 0.65 ± 0.03, *P* < 0.01). However, the expression of IL-17A in the mica group was significantly decreased compared to that in the TNBS group (2.63 ± 0.64 vs. 6.93 ± 0.44, *P* < 0.01) (Table 8).

### 3.6. Statistical Analysis

The level of Ang II had a positive correlation with the colonic macroscopic damage score (*r* = 0.589, *P* < 0.05), high correlation with the colonic histologic damage score (*r* = 0.855, *P* < 0.01), moderate correlation with the level of IL-17A (*r* = 0.647, *P* < 0.05), and high negative correlation with IL-10 (*r* = 0.720, *P* < 0.01).

The levels of ACE2 and Ang (1-7) were negatively correlated with the colonic macroscopic damage score (ACE2: *r* = −0.631, *P* < 0.05; Ang (1-7): *r* = −0.880, *P* < 0.01), had a moderate negative correlation with the colonic histologic damage score (*r* = −0.600, -0.618, *P* < 0.05) and the level of IL-17A (*r* = −0.556, -0.518, *P* < 0.05), and had a high positive correlation with the level of IL-10 (*r* = 0.776, 0.769, *P* < 0.01).

## 4. Discussion

Ulcerative colitis is a nonspecific chronic inflammatory disease with unclear pathogenesis. The major causes of ulcerative colitis are genetic, immune, environmental factors, and microorganisms. So far, no effective treatment has been developed.

RAS systems have an important role in a variety of pathophysiological processes. Ang II is the main active substance in the RAS system. Ang II is a proinflammatory substance, which induces the expression of NF-*κ*B, p38MAPK activation, and generates a variety of inflammatory factors, such as IL-6 and IL-17 [12]. Ang II can also have a direct effect on the AT1 receptor on the intestinal cell membrane [13]. It can stimulate the MAPK signaling pathway through the phosphorylation cascade reactions, such as family activate downstream transcription factor that induces the expression of inflammatory factors [14]. Ang II is also part of the ACE-Ang II-AT1R axis, which is mediated through the AT1 receptor. Ang II expression can also lead to blood vessel shrinkage and boot string blood pressure.

Besides the heart, kidney, and brain, RAS is also expressed in the gastrointestinal [15]. Katada et al. [16] found that the expression of Ang II and AT1R in the experimental colitis mice is significantly increased, while the inflammatory response in mice without AT1R is significantly reduced. In the endothermic mice, peroxides significantly increased after Ang II activation, causing endothelial dysfunction and the intestinal tissue ischemia. When mice were treated with Ang II receptor antagonist, the intestinal tissue after ischemia and damage was significantly reduced [17].

Ang (1-7), which has vasodilatation and anti-inflammatory effects, is produced from Ang II. Angiotensin-converting enzyme 2 (ACE2), a new member of the RAS family, is a new ACE homolog. The structures of ACE and ACE2 are similar, but their biological activity is significantly different. ACE2 can increase anti-inflammatory factors such as IL-10 and generate Ang (1-7) of Ang II decomposition. Ang (1-7) is a 7-peptide substance that inhibits inflammation by promoting vasodilation, antioxidative stress, and inflammation alleviation [18]. Ang (1-7) is considered to be one of the endogenous Ang II antagonists, which can increase blood flow in the kidney, brain, mesenteric vascular endothelial, and multiple organ tissues, promoting the vasodilation and having a role in the angiogenesis organization [19]. A previous study [12] showed that inflammation of the intestine increases Ang II and promotes secretion of a variety of proinflammatory factors (TNF-*α*, IL-17A, TGF-*β* 1, etc.) from epithelial cells in the focal area. These factors further aggravate the inflammatory response causing damage to the intestinal tissue.

Khajah et al. [20] found that the levels of Ang II, ACE2, and Ang (1-7) were significantly increased in the DSS-induced colitis mice. When the exogenous Ang (1-7) was given, the colonic mucosa damage and ulcer improved. Moreover, Rodrigues et al. [21] showed that colonic mucosa damage in the DSS-induced colitis mice was alleviated after treatment with exogenous Ang (1-7). In contrast, the colon tissue damage was significantly aggravated after Ang (1-7) was blocked by antagonist A779, Ang (1-7) specific receptor. Furthermore, in our previous study, we found an ACE2-Ang (1-7)-MAS axis in the small intestinal mucosa of rats. The expression of ACE2, Ang (1-7), and MAS in the ACE2 group was significantly higher than that in the model group, while the expression of Ang II was significantly downregulated. Gross, pathological, and electron microscopic results showed that the injury of intestinal mucosa was significantly reduced compared with that of the model group. The expression of ACE2, Ang (1-7), and MAS in the MasR antagonistic group was significantly lower than that in the model group. In contrast, the expression of Ang II was significantly upregulated in the MasR antagonist group. Compared with the model group, the injury of intestinal mucosa in the MasR antagonistic group was more severe than that in the model group, which suggested that the ACE2-Ang (1-7)-MAS axis had a protective effect on NSAID-related small intestine injury. ACE2-Ang (1-7)-MAS may be a potential target for the prevention and treatment of NSAID-related small intestinal injury.

ACE2, which has the catalytic activity of carboxypeptidase, is less expressed in normal organisms than ACE, which is about 1/10 of ACE. Ang I and Ang II are the main substrates of ACE2. On the one hand, ACE2 can reduce the vasoconstriction and oxidative stress of Ang II by downregulating the level of Ang II; on the other hand, it can further antagonize the effect of Ang II by increasing the production of vacillating substance Ang (1-7) [22]. Ang (1-7) is the Ang II endogenous inhibitor. Previous studies have found that the level of ACE2 increases in many diseases, such as myocardial infarction, diabetes, kidney disease, and cirrhosis [23], thus suggesting that the RAS system is activated in the pathological state and the increase of ACE2 may be a stress response. In this study, we established colitis in mice using TNBS. These mice showed poor food intake, decreased activity, weight loss, loose stool, positive stool occult blood, and partial bloody stool. Moreover, the colonic tissue showed intestinal wall thickening, ulcer, necrosis, and other visible damage. In the TNBS group and the mica group, the score of macroscopic damage and histological damage was significantly higher compared to that in the control group. The expression level of IL-17A, Ang II, ACE2, and Ang (1-7) was significantly higher than that of the control group, indicating that Ang II, ACE2, and Ang (1-7) participated in the pathogenesis of colitis. High expression of Ang II can cause vasoconstriction of the intestinal mucosa, decrease of tissue blood supply, and damage of intestinal barrier function. At the same time, Ang II can also be used as an inflammatory factor to upregulate the level of IL-17A, further aggravating the inflammatory response, which may be an important pathogenesis of experimental colitis in mice.

Mica is a kind of natural layered mineral crystal, which is one of the silicate mineral drugs. Its main components are silicon dioxide and alumina. It has special physical properties, such as adsorbability, expansibility, plasticity, and ion-exchange property, so it can be adsorbed on the mucosal surface and enhance the function of the mucosal barrier. Previous studies indicated that mica could be used to treat diarrhea in children [24]. Mica can directly affect the mucosal surface, adsorb proinflammatory cytokines, promote mucus secretion, etc., which, in turn, reduce inflammatory cell infiltration and strengthen the mucosal barrier function [7]. Some studies [19, 25] have suggested that mica can also absorb proinflammatory factors, reduce intestinal mucosal permeability, and antagonize intestinal bacterial translocation. In our previous study [26], we found that the damage of colonic tissue in experimental colitis mice was significantly reduced after the intervention of mica microparticles, thus suggesting that mica can reduce the damage of colonic tissue in colitis mice.

In this study, we found that mica reduced weight loss, improved fecal characteristics, and reduced stool in the blood compared with those of the TNBS group. Moreover, the disease activity index was significantly lower than that of the TNBS group. The scores of macroscopic damage and histological damage were significantly reduced compared with those of the TNBS group, suggesting that mica intervention could significantly reduce the intestinal damage of the mice. Furthermore, we found that the expression of ACE2, Ang (1-7), and IL-10 in the colon tissue of the mica group was higher than that of the TNBS group. In comparison, the expression of Ang II and IL-17A was significantly lower, suggesting that mica can reduce inflammation and intestinal damage. Moreover, the correlation analysis showed that the expression of Ang II was positively correlated with the macroscopic damage score, histological damage score, and IL-17A and negatively correlated with IL-10. Ang II high expression can induce intestinal mucosa vasoconstriction, reduce tissue blood supply, and cause intestinal barrier function injury.

The expression of ACE2 and Ang (1-7) was negatively correlated with the macroscopic damage score, histological damage score, and IL-17A, but positively correlated with IL-10, showing that Ang II in experimental colitis mouse colon injury plays an essential role in the process. All these data indicate that the RAS system is involved in the development of experimental colitis in mice. During an early stage of colon injury, the body's self-defense mechanism is activated, ACE2 Ang-(1-7)-Mas axis expression level feedback to rising, to a certain extent can adversely affect Ang II proinflammatory role, chemotaxis raise of inflammatory cells and plays a role of negative regulating the release of inflammatory factor, its specific mechanism remains to be further researched. Mica on experimental colitis mouse colon injury has a protective effect; its mechanism in addition to adsorbing the inflammatory factor may also increase ACE2-Ang (1-7)-Mas axis, restrain Ang II 1 expression, and reduce inflammation reaction.

To sum up, our data suggested mica can alleviate TNBS-induced colitis in mice by regulating the inflammation process. Mica reduces Ang II and IL-17A and increases ACE2, IL-10, and Ang (1-7).

## Figures and Tables

**Table 1:** Disease activity index.

Weight loss (%)	Stool consistency^∗^	Occult/gross bleeding	Score
(-)	Normal	Normal	0
1-5			1
5-10	Loose	Guaiac (+)	2
11-15			3
>15	Diarrhea	Gross bleeding	4

The disease activity index = (combined score of weight loss, stool consistency, and bleeding)/3. ^∗^Normal stools = well-formed pellets; loose = pasty stools which do not stick to the anus; diarrhea = liquid stools that stick to the anus.

**Table 2:** Macroscopic damage score.

Macroscopic damage	Score
Normal colonic mucosa	0
Local hyperemia, no ulcer	1
Single ulcer, no obvious inflammation	2
A single ulcer with inflammation	3
Two or more ulcers with inflammation	4
Large ulcer with inflammation	5

**Table 3:** Histological grading of colitis.

Feature graded	Grade	Description
Inflammation	0	None
	1	Slight
	2	Moderate
	3	Severe

Extend	0	None
	1	Mucosa
	2	Mucosa and submucosa
	3	Transmural

Regeneration	4	No tissue repair
	3	Surface epithelium not intact
	2	Regeneration with crypt depletion
	1	Almost complete regeneration
	0	Complete regeneration or normal tissue

Crypt damage	0	None
	1	Basal 1/3 damaged
	2	Basal 2/3 damaged
	3	Only surface epithelium intact
	4	Entire crypt and epithelium lost

Percent involvement	1	1-25%
	2	26-50%
	3	51-75%
	4	76-100%

**Table 4:** Weight loss (%).

Group	*N*	D2-1	D3-2	D4-3
Control group	10	1.35 ± 1.00	4.41 ± 3.39	6.31 ± 2.88
TNBS group	9	−15.19 ± 4.04^▲^	−17.69 ± 4.97^▲^	−17.63 ± 6.45^▲^
Mica group	9	−6.30 ± 1.84^▲■^	−12.87 ± 4.99^▲■^	−11.29 ± 5.30^▲■^
*F*		176.144	155.118	260.946

^▲^ *P* < 0.01 vs. control; ^■^*P* < 0.01 vs. TNBS.

**Table 5:** Disease activity index $\left(\overset{¯}{x\;}\pm s\right)$.

Group	*N*	D2-1	D3-2	D4-3
Control group	10	0.00 ± 0.00	0.00 ± 0.00	0.00 ± 0.00
TNBS group	9	10.33 ± 1.50^▲^	11.33 ± 1.21^▲^	10.01 ± 2.04^▲^
Mica group	9	6.33 ± 1.36^▲■^	7.5 ± 1.97^▲■^	7.1 ± 2.48^▲■^
*F*		118.226	111.460	47.532

^▲^ *P* < 0.01 vs. control; ^■^*P* < 0.01 vs. TNBS.

**Table 6:** Macroscopic and histological damage score $\left(\overset{¯}{x}\pm s\right)$.

Group	*N*	Macroscopic damage score	Histological damage score
Control group	10	0.00 ± 0.00	1.33 ± 1.03
TNBS group	9	4.00 ± 0.89^▲^	8.17 ± 2.99^▲^
Mica group	9	2.00 ± 1.54^▲■^	3.83 ± 1.47^▲■^
*F*		22.50	17.64

^▲^ *P* < 0.01 vs. control; ^■^*P* < 0.01 vs. TNBS.

**Table 7:** The expression of Ang II, ACE2, Ang (1-7), and IL-10 in colon tissue.

Group	*N*	Ang II	ACE2	Ang (1-7)	IL-10
Control group	10	2.16 ± 0.41	2.04 ± 0.29	0.13 ± 0.14	2.71 ± 0.78
TNBS group	9	4.83 ± 2.11^▲^	3.50 ± 0.55^▲^	1.04 ± 0.56^▲^	4.88 ± 0.83^▲^
Mica group	9	2.33 ± 0.52^▲■^	5.13 ± 1.84^▲■^	2.00 ± 0.60^▲■^	9.04 ± 2.62^▲■^
*F*		11.33	8.19	21.7	22.71

^▲^ *P* < 0.05 vs. control; ^■^*P* < 0.05 vs. TNBS.

**Table 8:** The level of IL-17A in colon tissue $\left(\overset{¯}{x}\pm \text{s},\text{pg}/\text{ml}\right)$.

Group	*N*	IL-17A
Control group	10	0.65 ± 0.03
TNBS group	9	6.93 ± 0.44^▲^
Mica group	9	2.63 ± 0.64^▲■^
*F*		26.39

^▲^ *P* < 0.01 vs. control; ^■^*P* < 0.01 vs. TNBS.

## Data Availability

All data, models, and code generated or used during the study appear in the submitted article.

## Abbreviations

IBD: Inflammatory bowel disease
RAS: Renin-angiotensin system
TNBS: 2,4,6-Trinitrobenzenesulfonic acid solution
Ang II: Angiotensin II
ACE2: Angiotensin-converting enzyme 2
Ang (1-7): Angiotensin 1-7.
